# Analyzing the Relationship between the Chemical Composition and the Surface Finish of Alnico Alloys in EDM

**DOI:** 10.3390/ma16206765

**Published:** 2023-10-19

**Authors:** Piotr Młynarczyk, Damian Bańkowski, Bartłomiej Szwed

**Affiliations:** Department of Metal Science and Manufacturing Processes, Faculty of Mechatronics and Mechanical Engineering, Kielce University of Technology, al. Tysiaclecia Panstwa Polskiego 7, 25-314 Kielce, Poland; piotrm@tu.kielce.pl (P.M.); bszwed@tu.kielce.pl (B.S.)

**Keywords:** electrical discharge machining (EDM), white layer, alnico, roughness, surface finish

## Abstract

The purpose of this study was to determine whether the chemical compositions of Alnico alloys had any effects on the electrical discharge machining (EDM) performance and the surface finish. This article compares the behavior of three different Alnico alloys in electrical discharge machining. The experiments were conducted under different conditions using a BP93L EDM machine (ZAP BP, Końskie, Poland), applying an additional rotary motion to the electrode. A Box–Behnken experimental design was employed to analyze the influence of three factors, i.e., the spark current, the pulse-on time, and the pulse-off time, at three levels for three Alnico alloys. The material removal rate (MRR) was calculated for the different process parameters. After the EDM, the surface roughness was studied using a Talysurf CCI Lite non-contact profiler (Taylor–Hobson, Leicester, UK). The next step of the experiments involved preparing metallographic specimens to be observed by means of scanning electron microscopy (SEM) and optical microscopy (OM). Measurements of the nanohardness were also performed. The experimental data were then analyzed using Statistica software version 10 (64-bit) to determine and graphically represent the relationships between the input and output parameters for the three Alnico alloys. The chemical compositions of the Alnico alloys affected the thickness of the white layer (higher cobalt content, lower white layer thickness) and the material removal rate. The higher the cobalt content, the thinner the white layer and the lower the material removal efficiency. Moreover, the cobalt content in Alnico alloys influenced the shape of the precipitates; these ranged from spheroidal (13% Co) to mix-shaped (21.3% Co) to flake-shaped (32.2%). The hardness of the resulting white layer was 874 HV at10 mN.

## 1. Introduction

Electrical discharge machining (EDM) is known to be particularly suitable for materials that are difficult to machine in a conventional way [1,2,3,4,5,6,7]. This process is used, for example, to produce tools and molds or to shape very brittle materials [8,9,10,11].

The physical phenomena occurring in EDM have been studied extensively for years. The erosion processes take place as a result of the large amounts of energy required to apply voltage pulses between the workpiece and the tool [12,13,14,15]. The process physics is based on local discharges between the workpiece and the tool [16,17,18,19]. The process parameters are mainly the electrical energy parameters, i.e., voltage and current. The output parameters can be predicted by determining the relationship between the discharge current parameters and the pulse duration. The EDM performance is largely dependent on the duration of the spark (pulse-on time) and the period between sparks required to clean out the eroded debris (pulse-off time) [20,21,22,23].

Published studies on the subject [3,4,11,13,24] state that the temperature in EDM may locally reach over 12,000 degrees Kelvin. It is a high-energy process. It should be noted that material loss occurs as a result of heating the material to a very high temperature, followed by its evaporation in the plasma channel [4,5,14]. It can be expected that unsuitable machining conditions may lead to unintended behavior of erosion products on the workpiece. The phenomenon, known as the formation of a characteristic white layer [21], is thoroughly discussed in the literature. The white layer can be seen by performing metallographic examinations that involve etching. There are many studies dealing with the effects of the machining parameters on the thickness of the white layer.

The word Alnico is an acronym referring to a family of iron-based cast or sintered materials containing mainly aluminum, nickel, and cobalt, hence the name Al–Ni–Co. The other elements found in Alnico materials include copper and titanium [24]. Altogether, there are 29 Alnico grades, of which 17 are produced by casting (C), 10by sintering (S), and two by bonding (B) [24,25,26,27,28,29]. The commercial names of Alnico alloys include Alnico, Columax, Alcomax 3SC, Alni, Hycomax, and Ticonal [29]. It is a group of materials with excellent magnetic properties and high hardness. The high hardness is related to high-energy defects in the crystal lattice structure, which makes machining very difficult. Milling and turning are practically impossible because of material cracking and spalling [28]. Unconventional machining, i.e., electrical discharge machining (EDM), can produce complex geometries without the use of any mechanical forces [30,31].

There are many published research findings regarding the influence of EDM parameters on the surface texture (roughness) [23,32,33,34] or the material removal rate [12,35]. Świercz [36] examined the thickness of the white layer forming in EDM as a function of current, pulse-on time, and pulse-off time.

The novelty and scope of the present study is about the influence of the chemical composition (of the Alnico alloy), its cobalt content, current intensity, pulse-on time, and pulse-off time on the electrical discharge machining performance and the surface quality. The preliminary findings of this research show that chemical composition is very important during electrical discharge machining. Even the same grade of alloy with different chemical compositions may have different effects on the EDM process. The major purpose of this study was to link the occurrence of a white layer, typical of EDM, and its thickness with the chemical composition of the Alnico alloys to assess the EDM performance and the surface quality (surface roughness). The relationships between the input parameters (spark current, pulse-on time, and pulse-off time) and the output parameters were represented graphically.

## 2. Materials and Methods

Three Alnico alloys differing in chemical composition were processed by electrical discharge machining. The specimens of the three materials, i.e., Alnico 2, 5, and 8, were in the form of rods with a length of 18 mm and a diameter of 5 mm. The alloys were selected based on the analysis of their availability and chemical compositions. They are characterized by different magnetic properties and are therefore used in different applications [37]. The experiments performed for three different Alnico alloys helped predict, in a utilitarian way, the optimal machining parameters for each alloy considered. The selection of materials for the research was guided primarily by the linear change in the content of cobalt (increasing approximately every 9%). The contents of cobalt, being about 13%, 21.3%, and 32.2%, respectively, were integrated into the experimental design by assuming the values −1, 0, and 1.

The experiments involved analyzing the influence of the chemical composition of each material on the EDM performance and the specimen surface quality. The machining was performed under different conditions. The tests aimed to analyze how the chemical composition of a material and specific process parameters affected the electrical discharge machining performance. The Alnico alloys selected for the tests differed in their content of cobalt.

As in the case of steel, the main factor influencing hardenability is the chemical composition, i.e., the content of carbon and the alloying elements and additives. In addition, the grain size and homogeneity of austenite, as well as the presence of other undissolved particles, also influence hardenability. All alloying elements except for cobalt increase hardenability. The presence of Mn, Mo, Cr, and B seems to be the greatest, depending on the solubility of each element in austenite for stainless steel. However, cobalt may also reduce the hardenability, as is the case with stainless steel. This occurs by increasing the critical cooling rate, i.e., the lowest cooling rate, allowing a martensitic structure to be obtained; it isrelated to the durability of subcooled austenite until its transformation into martensite begins. From the literature [38], it is evident that cobalt is responsible for the lower hardenability of iron-based alloys, which may have some effect on the EDM performance and the output parameters.

**Figure 1:**
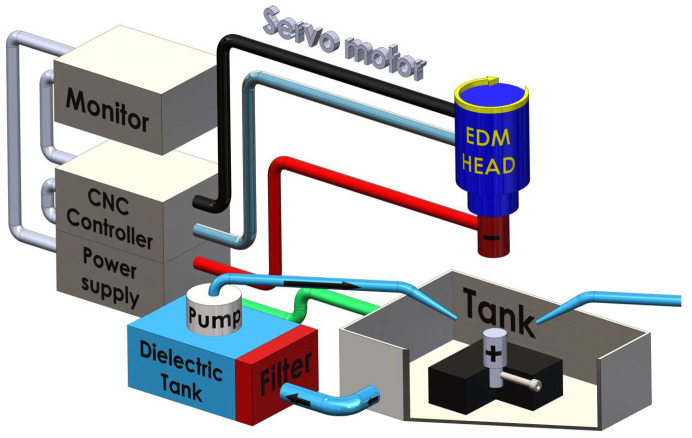
Schematic diagram of the test setup.

The chemical compositions of the materials tested were determined with a JEOL JSM-7100F field emission scanning electron microscope (JEOL, Tokyo, Japan). The results are provided in Table 1. The tests were carried out using the setup illustrated in Figure 1.

The tests were performed at a maximum spark current of 35 A on a 3 kW BP93L EDM machine (ZAP BP, Końskie, Poland) with kerosene employed as the dielectric fluid. Dielectric stability was achieved through forced circulation filtration. The aim of experimental research should be to obtain information regarding the relationship between the input and output variables. When research is planned, the costs and time of experiments need to be taken into account. The number of input quantities or input values should be small. If the scope of experiments is too narrow, reliable conclusions may not be drawn. Statistica version 10 (64bit) (TIBCO Software Inc., Tulsa, OK, USA) offers ready-to-use experimental designs (DoE module) built into the software. In this study, the 3 × 3 Box–Behnken experimental design was selected based on the process parameters. The Box–Behnken approach helped reduce the number of experiments needed to be performed from 27 to 15 and, consequently, reduced the costs and time of testing. The Box–Behnken design of experiments with three factors and three levels was used to link the process parameters with the surface finish parameters. The input parameters were the spark current, *I*, the pulse-on time, *t_on_*, and the pulse-off time, *t_off_.* Table 2 shows the EDM parameters used in the experiments for the three grades of Alnico alloys, the chemical compositions of which are provided in Table 1.

The specimens to be examined using the optical and scanning electron microscopes were prepared first by embedding the material tested in Technovit 5000 (Kulzer GmbH, Hanau, Germany) conductive resin, then sequential grinding it on a Presi Minitech 263 (Presi, Eybens, France) with silicon carbide (SiC) abrasive paper (120–2500), and finally polishing it on a Struers LaboPol-5 (Struers, Copenhagen, Denmark) using 1 μm polycrystalline diamond suspension and SUPRA cloth (Microdiamant, Kempen, Germany).

The surface of each specimen was analyzed using a JEOL JSM-7100F(JEOL, Tokyo, Japan) field emission scanning electron microscope equipped with an Oxford Instruments X-max energy dispersive spectrometer (EDS) (Oxford Instruments, Bucks, UK) detector and a back-scattered electron (BSE) detector, operating at a magnification of 10–1,000,000× and an accelerating voltage of 100 V−30 kV. The observations were made at a resolution of 1.2 nm or 3 nm. The voltage was 30 kV and 1kV, respectively. During the examinations, the current intensity was 1 nA, and the tool-to-work distance was 10 mm. The results were analyzed using Aztec version 1.2.

The specimen mass was measured using a RADWAG AS 160/X (Radwag, Radom, Poland) analytical balance to the nearest ±0.1 mg.

The 2D and 3D surface roughness parameters were determined with a Taylor–Hobson Talysurf CCI Lite non-contact 3D profiler (Taylor–Hobson, Leicester, UK). The area measured was 1.6 mm x 1.6 mm. The surface texture analysis was conducted using a Gaussian filter (cut-off 0.8 mm).

The microstructural analysis to study the material morphology and the thickness of the white layer was conducted using a Nikon Eclipse MA200 optical microscope (Nicon, Minato-ku, Japan) equipped with NIS 4.20-Elements Viewer imaging software version XT 6.8.

The relationships between the input and output parameters were visualized using Statistica 10. After EDM, the hardness of each material was measured with an Innovatest Nexus 400 tester (Innovatest, Maastricht, The Netherlands).

## 3. Results and Discussion

First, the EDM performance was studied. The mass of each specimen was measured before and after the test. Before weighing, the specimens were placed in an ultrasonic tank to be cleaned using alcohol and air-dried to remove the eroded debris. The tests involved machining the material to a depth of 5 mm under the conditions determined in the 3 × 3 Box–Behnken experimental design (Table 3). After the desired depth was reached, the electrode was retracted automatically. The pulse duration time was measured using a stopwatch.

The EDM performance was evaluated based on the MRR (cm^3^/s):

(1) $$\mathrm{MRR}=\frac{\left(m_{1}-m_{2}\right)\cdot 1000}{\rho \cdot t}$$

where: *m*_1_—specimen mass before EDM, [g],
*m*_2_—specimen mass after EDM, [g],
*ρ*—density of Alnico = 7.3 [g/cm^3^],
*t*—pulse duration time, [s].

**Figure 2:**
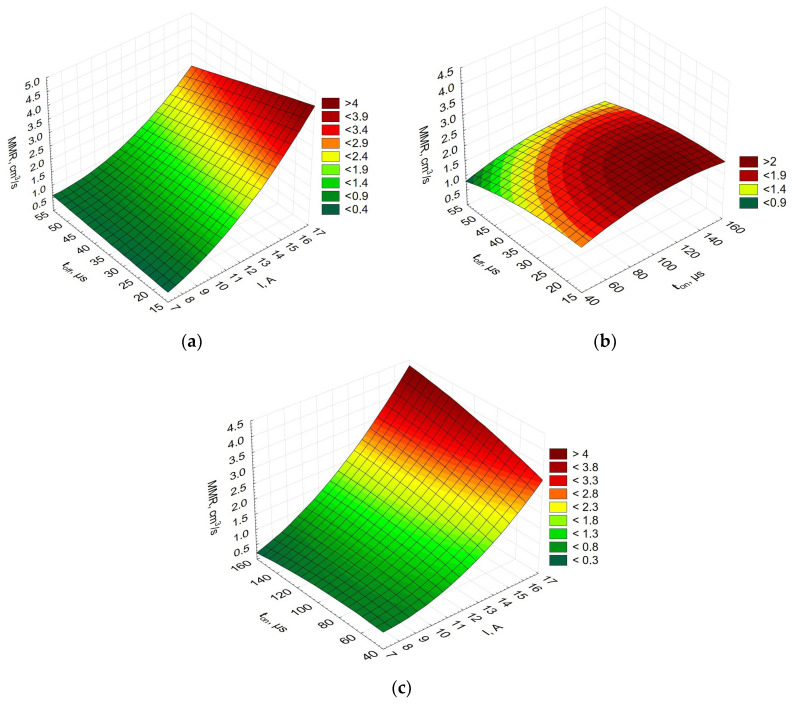
MRR for Alnico 2 (**a**) *t_off_* vs. *I*, and (**b**) *t_ofvf_* vs. *t_on_*,|(**c**) *t_on_* vs. *I*—for Alnico 2.

The experimental data were analyzed using the Statistica 10 software to determine how the EDM performance was influenced by each factor for all the Alnico alloys. Figure 2 shows the effects of the selected factors on MRR.

Figure 2 shows the process performance expressed by MRR established for Alnico 2, where pulse-off time, *t_off_*, is plotted as a function of current, *I*, pulse-off time, *t_off_*, is plotted as a function of pulse-on time, *t_on_*, and pulse-on time, *t_on_*, is plotted as a function of current, *I*. Since the results obtained for Alnico 5 and Alnico 8 were similar, they are not included here. As can be seen from Figure 2, the volume of the material removed or MRR was most affected by current. If both the current and the pulse-on time are increased, large mass loss is observed. Also, from Figure 2a, it can be concluded that a shorter pulse-off time at high current results in greater erosion intensity. This is directly connected with the coefficient of discharge *α.*

(2) $$\alpha =\frac{t_{on}}{t_{on}+t_{off}}$$

The higher the values of the coefficient α, the more frequent the discharges. As a result, the machining time can be shorter. However, if the values of the coefficient of discharge are too high, the removal of the eroded debris may not be efficient. Consequently, the gap between the tool and the workpiece is blocked, and some undesirable short circuits occur locally.

In EDM, changes in the spark current are mainly responsible for changes in the surface topography. Dabade and Karidkar [39] show that the irregular discharge energy generated by servo voltage and pulse-on time has a significant impact on roughness and geometric errors. At lower values of the spark current, *I*, the erosion of particles is slower. The debris is removed in very small amounts by evaporation. The higher the spark current, the higher the power of a single discharge and, consequently, the greater the erosion of the material particles (larger vapor drops), especially when the pulse-on time is longer. From a physical point of view, longer pulse-on times, *t_on_*, mean longer discharge times and longer plasma channel formation times. Under such conditions, large, deep valleys form. Short discharge times contribute to deeper but smaller valleys. Longer pulse-off times result in lower values of the spatial parameters; the flushing of the debris from the cutting zone is more efficient. Shorter pulse-off times, *t_off_*, lead to the inefficient removal of solid particles, which may cause short circuits and surface damage.

The lowest surface roughness was observed at a very low spark current. However, small energies of single discharges are responsible for the lower effectiveness of the processes. The removal of the eroded material takes much longer. It is, therefore, recommended that the electrical discharge machining process should be performed in two or even three stages. The first stage requires high power (high spark current, *I*, and high voltage, *U*), long pulse-on times, and short pulse-off times. The spark current and the time parameters are reduced gradually with every next step, depending on the surface quality requirements.

**Figure 3:**
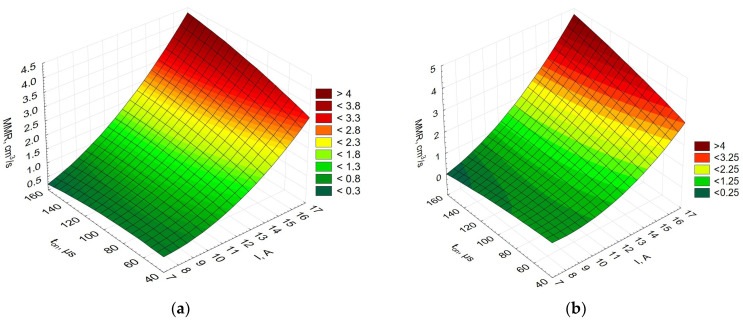

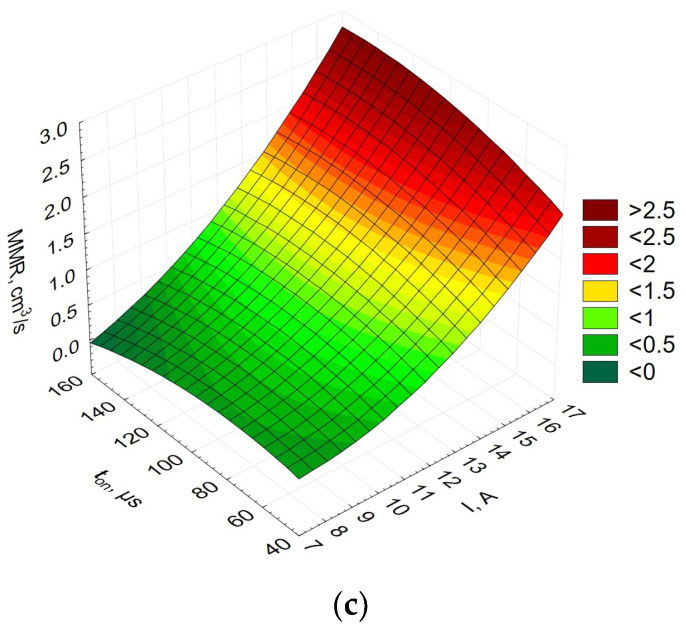
EDM performance expressed by MRR for (**a**) Alnico 2, (**b**) Alnico 5, and (**c**) Alnico 8.

Figure 3 compares the EDM performance for the three materials studied, i.e., Alnico 2, Alnico 5, and Alnico 8; it is expressed as MRR versus current, *I*, versus pulse-on time, *t_on_*.

Figure 3 shows that the EDM performance (MRR) is identical for all three alloys (Alnico 2, 5, and 8). An increase in current causes an increase in the energy density and faster erosion of the workpiece. Large volumes of material evaporate, which is not observed at much lower currents and shorter pulse durations. 

**Figure 4:**
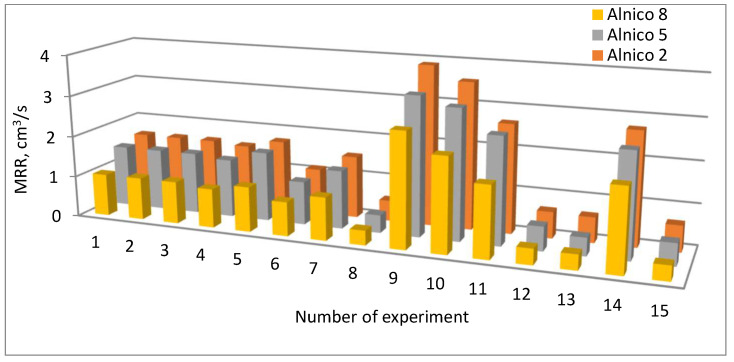
MRR for Alnico 2, Alnico 5, and Alnico 8.

Figure 4 compares the MRR in all the 15 EDM experiments performed for the three alloys.

The research data confirm that the best EDM performance (highest material removal rate) was achieved for Alnico 2, while the lowest for Alnico 8. The difference in the performance was due to the difference in their chemical composition (content of cobalt). This is particularly true when rapid thermal and physical changes take place because the presence of cobalt may reduce the material’s hardenability (its ability to transform rapidly into martensite with no diffusion).

The material removal rate was much slower, i.e., 34–76% slower, for Alnico 8 than for Alnico 2,depending on the process parameters. For Alnico 5, the process occurred more slowly (2–40%) than for Alnico 2. The difference in MRR for Alnico 5 and 8 reached 15–35%. This might have also been due to the formation of the so-called white layer, which was observed on different specimens machined in different conditions. The white layer was reported to be thicker for Alnico 2 than for Alnico 5 or 8.

The next step of the experiments involved determining the basic height (amplitude) and hybrid surface texture parameters. The two- and three-dimensional height parameters considered were the arithmetic mean roughness (*Ra*) and the mean summit curvature (*Ssc*) [40,41,42]. The hybrid parameters, on the other hand, were the summit density (*Sds*), the mean summit curvature (*Ssc*), and the root mean square surface slope (*Sdq*) [40,41,42].

**Figure 5:**
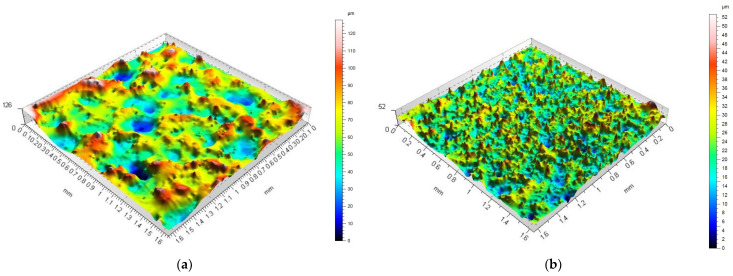
Isometric views of the specimen surface after EDM at (**a**) high-energy discharges; (**b**) low-energy discharges.

Figure 5 shows isometric views of the surface after roughing and finishing for Alnico 2.

The observations aimed to determine the highest peaks, the deepest valleys, and the number of summits. EDM with high-energy discharges (at high current and long pulse-on time) produces surfaces with a much smaller number of peaks and valleys, but their heights and depths are higher than those obtained with low-energy discharges (low current and short pulse-on time). Peaks were reported to have much bigger diameters when there was a smaller number of peaks per unit area. By analogy, the diameters were smaller for surfaces with more numerous peaks.

Some of the measurement data for experiments given in Table 3 were represented graphically using Statistica10 software to illustrate the relationships between the input and output parameters. Three factors were considered, but the plots provided in the article show the effects of the pulse current and duration, as these produced the most significant changes.

**Figure 6:**
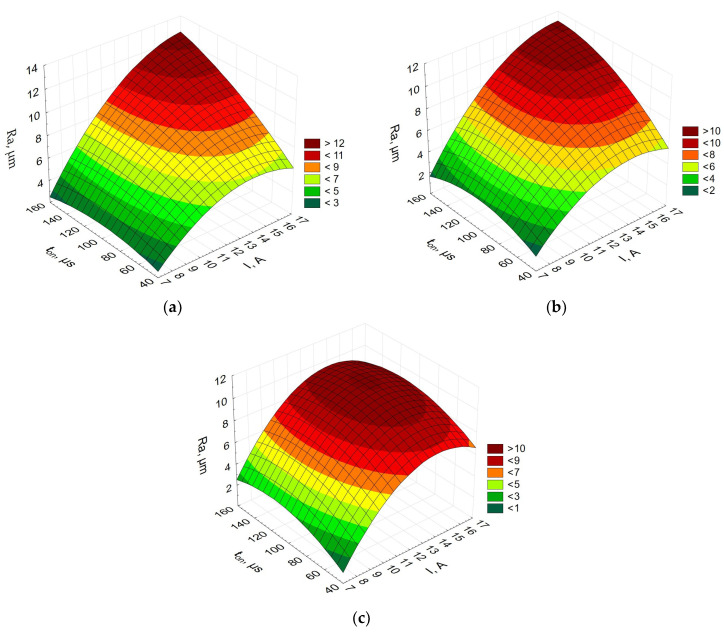
Arithmetic mean roughness (*Ra*) vs. current vs. pulse-on time for (**a**) Alnico 2, (**b**) Alnico 5, and (**c**) Alnico 8.

Figure 6 shows the relationship between the arithmetic mean roughness, the current, and the pulse-on time.

The relationships obtained for Alnico 2 and Alnico 5 look similar; the shorter the pulse-on time and the lower the current, the smaller the parameter *Ra*—the arithmetic mean roughness. However, for Alnico 8, with a higher content of cobalt, the relationships are different. As can be seen from Figure 5c, an increase in both the current and the pulse-on time leads to an increase in the arithmetic mean roughness. If the pulse-on time is more than about 120 µs, the parameter *Ra* decreases. The differences are probably due to the physical properties of the alloys. At high-density energy, the chemical composition will reduce the EDM performance and, consequently, the surface quality. Single high-energy discharges may not be able to remove the eroded debris effectively.

**Figure 7:**
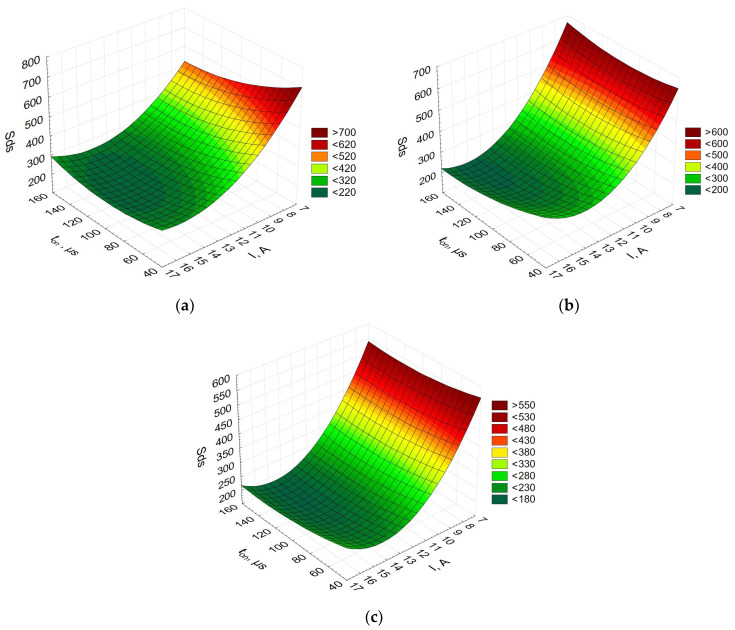
Summit density (*Sds*) vs. current vs. pulse-on time for (**a**) Alnico 2,(**b**) Alnico 5, and(**c**) Alnico 8.

Figure 7 depicts the relationship between the summit density, the current, and the pulse-on time.

The diagrams showing how the summit density was affected by the current and the pulse-on time were similar for the three Alnico alloys. This parameter indirectly determines the distances between the peaks in a given sampling region. The lower the value of the parameter *Sds*, the fewer the peaks. The current was observed to have the greatest influence on the number of peaks in EDM. The higher the current, the fewer the peaks. An increase in the pulse-on time also seems to be responsible for the smaller number of peaks.

**Figure 8:**
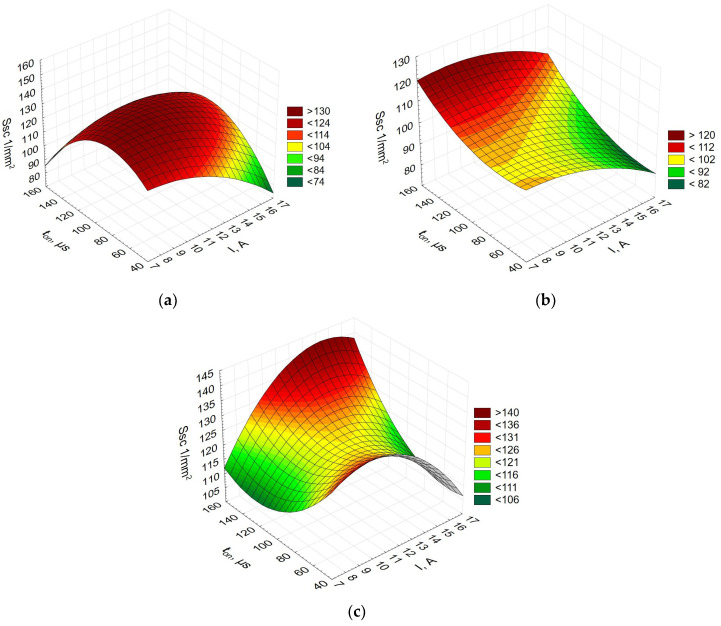
Mean summit curvature (*Ssc*) vs. current vs. pulse-on time for (**a**) Alnico 2, (**b**) Alnico 5, and (**c**) Alnico 8.

Figure 8 shows the relationship between the mean summit curvature, the current, and the pulse-on time.

From Figure 8, it is evident that for each alloy, the relationship between the parameter *Ssc*, the current, and the pulse-on time looks different. For Alnico 2, the highest values of *Ssc* were obtained at a pulse-on time of about 100 µs. For Alnico 5 and 8, the longest pulse-on time, t_on_, led to the highest mean summit curvature. The effect of the current on the parameter *Ssc* looks interesting. For Alnico 2 and 8, *Ssc* was low at both high and low values of the current. For Alnico 5, an increase in the current resulted in a decrease in the mean summit curvature.

**Figure 9:**
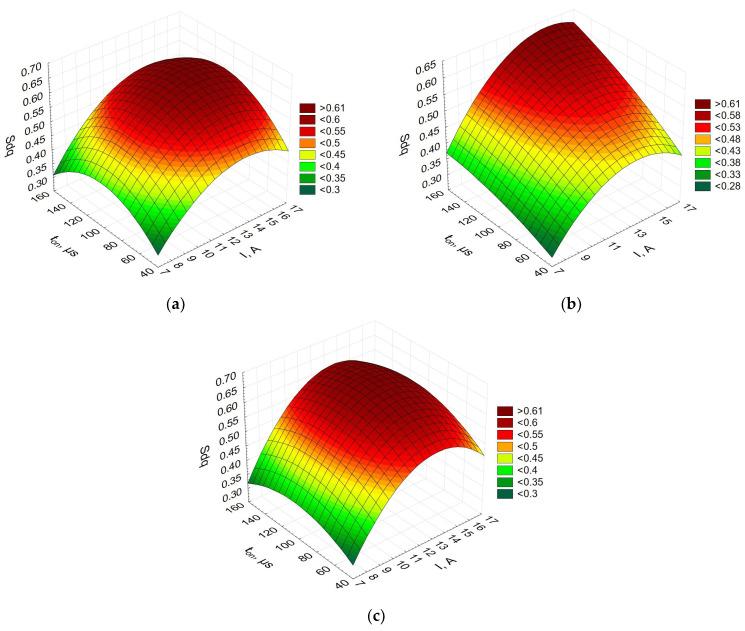
Root mean square surface slope (*Sdq*) vs. current vs. pulse-on time for (**a**) Alnico 2, (**b**) Alnico 5, and (**c**) Alnico 8.

Figure 9 displays the influence of the current and the pulse-on time on the root mean square surface slope (*Sdq*). 

The diagrams in Figure 9, showing the effects of the current and the pulse-on time on the root mean square surface slope, suggest that an increase in the current in EDM causes the parameter *Sdq* to increase. The root mean square surface slope (in mm/mm) was reported to be less dependent on the pulse-on time. The lowest values of the parameter *Sdq* were observed at shorter pulse-on times (about 40 µs).

One of the characteristic features of EDM is the occurrence of a white layer. In the study, it was revealed by etching the Alnico specimens with a 2% Nital solution. The white layer was observed using optical and scanning electron microscopy. It had a dendritic microstructure.

The metallographic examinations were conducted using central point specimen cross-sections. Each alloy subjected to electrical discharge machining (*I* = 12 A, *t_on_* = 100 µs; *t_off_* = 35 µs) was analyzed to determine the average thickness of the white layer and study the material changes caused by the rapid thermal, mechanical, and chemical processes. The mean results are shown in Table 4.

As can be concluded from Table 4, the chemical compositions of the Alnico alloys had some influence on the thickness of the white layer. The thickest white layer, ranging between 60 and 89 µm, was observed for Alnico 2. For Alnico 5, the layer had a thickness of 50–59 µm. Finally, the thickness of the white layer reported for Alnico 8 varied from 25 to 40 µm. The changes in the layer thickness seem to have resulted from the differences in the chemical composition. Alnico 5 and Alnico 8 contained 21.3% and 32.2% of cobalt, respectively. The content of cobalt in Alnico 2, however, was lower, reaching 12%. A higher amount of cobalt reduces the capability of an iron alloy to undergo martensite transformation. 

The formation of the white layer in EDM is due to the high temperature accompanying the evaporation of the material. The more energy is supplied (high current, long pulse time, and short pause time), the more heat is generated; as a result, larger losses of material and, consequently, thicker white layers are observed. However, when electrical discharge machining is performed at high energy densities, fewer deep, large craters form on the surface than in machining at low energy densities. Research in this area shows that the chemical composition of the workpiece has a significant impact not only on the material removal rate but also on the surface roughness, irrespective of the values of the process parameters. The chemical composition of the workpiece also affects the thickness of the white layer.

**Figure 10:**
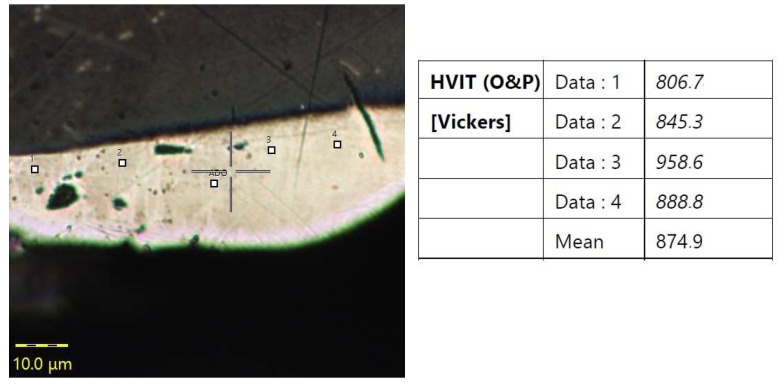
Microphotograph showing nanohardness measurement white dots points (1–4 and ADO-Adjust Deph Offset) for Alnico 8.

It was also essential to analyze whether the chemical compositions of Alnico alloys had any effect on the hardness of the white layer forming during EDM. Due to the small thickness of the white layers obtained, the nanohardness tests were conducted with an Anton Paar Step E600 (Anton Paar, Graz, Austria) (at a maximum load of 10 mN); the hardness of the base material was measured with an Innovatest Nexus 400 tester (Innovatest, Maastricht, The Netherlands) (at a load of 0.1 N) in the central point specimen cross-sections for each material machined at *I* = 12 A, *t_on_* = 100 µs; and *t_off_* = 35 µs. Figure 10 shows a nanohardness measurement of Alnico 8.

The measurement (white dots) shown in Figure 10 revealed that the nanohardness of the white layer formed in Alnico 8 was approx. 874 HV at 10 mN. Similar results were obtained for Alnico 2 and 5. From the measurement data obtained for the three Alnico alloys, it can be concluded that the nanohardness of the white layer was not dependent on the alloy grade and, consequently, its chemical composition.

The microhardness tests of the base material showed that Alnico 2 had an average hardness of approximately 616 HV; for Alnico 5, the average hardness was 537 HV; and for Alnico 8,it reached 652 HV. The results suggest that the hardness of the base metal depended on the phase transitions taking place during the solidification of the alloys. Based on the previous findings, additional research is being prepared to elucidate the impact of the phase composition on the properties of Alnico alloys. 

**Figure 11:**
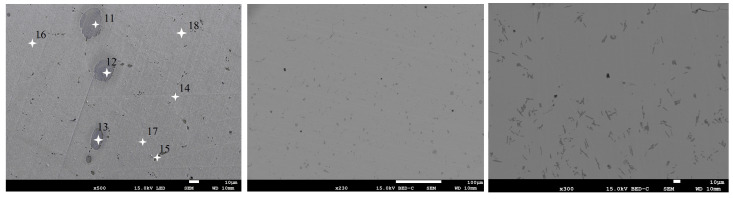
Precipitates in (**a**) Alnico 2 (magnification 500×;white stars indicate the location of the EDS measurement)), (**b**) Alnico 5 (magnification 230×), and (**c**) Alnico 8 (magnification 300×).

The SEM observations of Alnico 2, Alnico 5, and Alnico 8 revealed the occurrence of precipitates, as shown in Figure 11a, Figure 11b, and Figure 11c, respectively.

The chemical compositions of the precipitates present in the three alloys were analyzed. All the alloys had precipitates rich in aluminum, nickel, and copper. For example, the results obtained for Alnico 2, i.e., the compositions of the precipitates and the base metal (in Figure 11a, points 11–15, and 16–18, respectively) are given in Table 5.

The microphotographs in Figure 11 indicate that the precipitates predominant in Alnico 2 are spheroidal in shape; those present in Alnico 8 are generally flake-shaped; finally, Alnico 5 had mainly spheroidal precipitates and some flake-like precipitates. The chemical compositions of the Alnico alloys had an enormous influence on the shape and composition of precipitates (as compared to the composition of the matrix). Precipitates may be responsible for material crack formation and propagation and, consequently, for the mechanical properties of the material.

The precipitates depicted in Figure 11 are rich in aluminum, nickel, and copper. The precipitates differed from the base metal in chemical composition. Silicon was the only element whose content did not change substantially. From the analysis, it is clear that the precipitates are richer in such elements as aluminum, nickel, and copper, while they are poorer in iron and cobalt. On average, the precipitates contain 31.6% aluminum, 32.2% nickel, 8.8% copper, and 1.2% titanium, with iron reduced to 16.6% and cobalt to 9.0% by weight.

## 4. Conclusions

In the electrical discharge machining of Alnico alloys, it is necessary to take into account the chemical composition of the workpieces, particularly the percentage content of cobalt. A higher content of cobalt requires the use of lower energy densities to produce surfaces with an appropriately low surface roughness. Moreover, it is worth mentioning that as the energy of EDM processing decreases, less heat is released in the material, which results in a decrease in the thickness of the white layer. In the future, the research results may translate into EDM processing of other materials.

Electrical discharge machining performed at low voltages results in low arithmetic mean roughness (*Ra*) and high arithmetic mean summit curvature (*Ssc*).

Surfaces to be coated or painted need to be porous (extended); they can be produced by machining by EDM at high currents and long pulse-on times.

The chemical compositions of the Alnico alloys affected:

- the medium thickness of the white layer forming in EDM in the central point of experiments; for Alnico 2, Alnico 5, and Alnico 8, it was about 73.6 µm, 53.3 µm, and 33.0 µm, respectively;
- the material removal rate (MRR); for Alnico 2, it was the fastest, and for Alnico 8, it was the slowest;
- the shape of precipitates rich in aluminum, nickel, and copper; an increase in the content of cobalt in Alnico alloys causes a change in the shape of precipitates from spheroidal (Alnico 2 with 13.0% Co) to leaf-like (Alnico 8 with 32.2% Co). Alnico 5, containing about 21.3% Co, had both spheroidal and leaf-like precipitates.

Another observation made was a relationship between the thickness of the white layer and the arithmetic mean roughness. An increase in the thickness of the white layer led to an increase in the roughness parameter *Ra*.

The white layer formed on all specimens, irrespective of the chemical composition of the material used and the EDM process parameters employed.

The hardness of the white layer after EDM was approximately 874 HV at 10 mN; it was not dependent on the chemical composition of the Alnico alloy.

## Figures and Tables

**Table 1:** Chemical compositions of the Alnico alloys tested (wt.%).

Element	Al	Si	Ti	Fe	Co	Ni	Cu
Alnico 2	17.4	0.8	0.8	46.1	13.0	17.2	4.7
Alnico 5	14.7	1.6	-	46.2	21.3	13.3	2.8
Alnico 8	13.3	0.8	5.8	31.9	32.2	13.2	2.9

**Table 2:** Parameters and factors affecting the EDM process.

Variable	Value		
Voltage	230 V	230 V	230 V
Discharge current	8 A	12 A	16 A
Pulse-on time	50 µs	100 µs	150 µs
Pulse-off time	20 µs	35 µs	50 µs
Dielectric liquid	Kerosene		

**Table 3:** 3 × 3 Box−Behnken experimental design.

No.	Code Values			Actual Values			Alnico 2			Alnico 5			Alnico 8		
	*I*, A	*t_on_*, µs	*t_off_*, µs	*I*, A	*t_on_*, µs	*t_off_*, µs	MRR, cm^3^/s	*Ra,* µm	*Sds*	MRR, cm^3^/s	*Ra,* µm	*Sds*	MRR, cm^3^/s	*Ra,* µm	*Sds*
1	0	0	0	12	100	35	1.59	9.08	255	1.50	8.55	270	1.01	9.59	213
2	0	0	0	12	100	35	1.60	8.92	276	1.49	8.13	264	1.08	9.67	222
3	0	0	0	12	100	35	1.60	9.23	233	1.49	8.96	276	1.03	9.52	202
4	0	1	1	12	140	50	1.54	9.63	281	1.42	9.13	244	0.95	9.89	269
5	0	1	−1	12	140	20	1.73	9.33	249	1.68	9.15	235	1.09	9.73	200
6	0	−1	1	12	50	50	1.11	5.52	405	1.05	5.63	321	0.83	6.78	244
7	0	−1	−1	12	50	20	1.52	8.00	301	1.42	6.40	267	1.04	7.37	247
8	−1	0	1	8	100	50	0.50	4.39	636	0.43	3.91	549	0.36	4.15	524
9	1	0	−1	16	100	20	3.90	10.20	245	3.96	10.30	217	2.77	9.72	180
10	1	1	0	16	140	35	3.56	12.10	248	3.84	10.20	200	2.29	8.95	189
11	1	−1	0	16	50	35	2.65	7.65	258	2.60	6.60	280	1.73	8.16	243
12	−1	−1	0	8	50	35	0.64	4.06	562	0.59	3.74	562	0.39	4.18	459
13	−1	1	0	8	140	35	0.62	4.53	436	0.44	4.26	569	0.37	4.90	412
14	1	0	1	16	100	50	2.74	9.51	213	2.80	9.89	228	1.99	9.42	199
15	−1	0	−1	8	100	20	0.64	5.01	373	0.54	3.85	456	0.36	4.64	407

**Table 4:** The average thickness of the white layer forming in EDM.

	Alnico 2	Alnico 5	Alnico 8
white layer thickness, µm	73.6	53.3	33.0

**Table 5:** Chemical compositions (%) of the precipitates at the points marked in Figure 11a.

Spectrum Label	Al	Si	S	Ti	Fe	Co	Ni	Cu	Total
11	31.2	0.5			20.8	9.7	29.	8.2	100.0
12	33.8	0.5		1.5	11.1	8.2	34.9	10.0	100.0
13	30.9	0.5			19.8	9.9	30.5	8.4	100.0
14	31.1	0.5	2.3	0.8	15.2	9.3	32.3	8.5	100.0
15	31.2	0.6	0.7		15.8	9.3	33.7	8.7	100.0
16	18.5	0.8			45.4	12.9	17.9	4.5	100.0
17	18.5	0.9			45.2	12.6	18.0	4.8	100.0
18	18.3	0.9			45.2	12.7	18.0	4.9	100.0

## Data Availability

No publicly archived datasets are reported or used.

## References

[1] Singh R., Singh R.P., Trehan R. (2022). Surface integrity and accuracy based aspects in EDM of Cu-based SMA: An experimental investigation with microstructural analysis. Adv. Mater. Process. Technol..

[2] Abdudeen A., Abu Qudeiri J.E., Kareem A., Ahammed T., Ziout A. (2020). Recent advances and perceptive insights into powder-mixed dielectric fluid of EDM. Micromachines.

[3] Alam M.N., Siddiquee A.N., Khan Z.A., Khan N.Z. (2022). A comprehensive review on wire EDM performance evaluation. Proc. Inst. Mech. Eng. Part E J. Process Mech. Eng..

[4] Mao X., Almeida S., Mo J., Ding S. (2022). The state of the art of electrical discharge drilling: A review. Int. J. Adv. Manuf. Technol..

[5] Dąbrowski L., Paczkowski T. (2005). Computer simulation of two-dimensional electrolyte flow in electrochemical machining. Elektrokhimiya.

[6] Młynarczyk P., Krajcarz D., Bańkowski D. (2017). The selected properties of the micro electrical discharge alloying process using tungsten electrode on aluminum. Procedia Eng..

[7] Sahu A.K., Thomas J., Mahapatra S.S. (2021). An intelligent approach to optimize the electrical discharge machining of titanium alloy by simple optimization algorithm. Proc. Inst. Mech. Eng. Part E J. Process Mech. Eng..

[8] Chaudhari R., Vora J.J., Patel V., Lacalle L.L.d., Parikh D. (2020). Effect of WEDM process parameters on surface morphology of nitinol shape memory alloy. Materials.

[9] Farooq M.U., Anwar S., Kumar M.S., AlFaify A., Ali M.A., Kumar R., Haber R. (2022). A Novel Flushing Mechanism to Minimize Roughness and Dimensional Errors during Wire Electric Discharge Machining of Complex Profiles on Inconel 718. Materials.

[10] Ishfaq K., Anwar S., Ali M.A., Raza M.H., Farooq M.U., Ahmad S., Pruncu C.I., Saleh M., Salah B. (2020). Optimization of WEDM for Precise Machining of Novel Developed Al6061-7.5% SiC Squeeze-Casted Composite. Int. J. Adv. Manuf. Technol..

[11] Khan A.A., Ndaliman M.B., Zain Z.M., Jamaludin M.F., Patthi U. (2011). Surface modification using electric discharge machining. (EDM) with powder addition. Appl. Mech. Mater..

[12] Tosun N., Pihtili H. (2003). The Effect of Cutting Parameters on Wire Crater Sizes in Wire EDM. Int. J. Adv. Manuf. Technol..

[13] Kozak J., Ivanov A., Al-Naemi F., Gulbinowicz Z. EDM electrode wear and its effecton processes accuracy and process modelling. Proceedings of the 15th International Symposium on Electromachining.

[14] Kozak J., Gulbinowicz Z. The Mathematical Modeling and Computer Simulation of Rotating Electrical Discharge Machining. WCECS 2009. Proceedings of the World Congress on Engineering and Computer Science.

[15] Chaudhari R., Vora J.J., Prabu S., Palani I., Patel V.K., Parikh D. (2021). Pareto optimization of WEDM process parameters for machining a NiTi shape memory alloy using a combined approach of RSM and heat transfer search algorithm. Adv. Manuf..

[16] Klocke F., Lung D., Antonoglou G., Thomaidis D. (2004). The Effects of Powder Suspended Dielectrics on the Thermal Influenced Zone by Electro discharge Machining with Small Discharge Energies. J. Mater. Process. Technol..

[17] Sharma D., Hiremath S.S. (2021). Review on tools and tool wear in EDM. Mach. Sci. Technol..

[18] Oniszczuk-Świercz D., Kopytowski A., Nowicki R., Świercz R. (2023). Finishing Additively Manufactured Ti6Al4V Alloy with Low-Energy Electrical Discharges. Materials.

[19] Oniszczuk-Świercz D., Świercz R. (2023). Effects of Wire Electrical Discharge Finishing Cuts on the Surface Integrity of Additively Manufactured Ti6Al4V Alloy. Materials.

[20] Oniszczuk-Świercz D., Świercz R., Kopytowski A., Nowicki R. (2023). Experimental Investigation and Optimization of Rough EDM of High-Thermal-Conductivity Tool Steel with a Thin-Walled Electrode. Materials.

[21] Oniszczuk-Świercz D., Świercz R., Michna Š. (2022). Evaluation of Prediction Models of the Micro wire EDM Process of Inconel 718 Using ANN and RSM Methods. Materials.

[22] Chaudhari R., Shah Y., Khanna S., Patel V.K., Vora J., Pimenov D.Y., Giasin K. (2022). Experimental Investigations and Effect of Nano-Powder-Mixed EDM Variables on Performance Measures of Nitinol SMA. Materials.

[23] Singh S.K., Mali H.S., Unune D.R., Wojciechowski S., Wilczyński D. (2022). Application of Generalized Regression Neural Network and Gaussian Process Regression for Modelling Hybrid Micro-Electric Discharge Machining: A Comparative Study. Processes.

[24] Bańkowski D., Młynarczyk P. (2022). Influenceof EDM Process Parameters on the Surface Finish of Alnico Alloys. Materials.

[25] Bańkowski D., Młynarczyk P., Szwed B. (2023). Effects of EDM on the Chemical Composition and Microstructure of the Surface Layer of Alnico Alloys. Arch. Foundry Eng..

[26] Młynarczyk P., Bańkowski D., Spadło S., Ziarkowski P., Grzęda J. Non Destructive Testing of Alnico Alloys. Proceedings of the 31st International Conference on Metallurgy and Materials.

[27] Bańkowski D., Młynarczyk P., Spadło S., Sójka R., Klamczyński K. Tomographic Testing of Alnico Alloys. Proceedings of the 31st International Conference on Metallurgy and Materials.

[28] https://pl.wikipedia.org/wiki/Alnico.

[29] https://magnesy.pl/magnesy-alnico.

[30] Ablyaz T.R., Shlykov E.S., Muratov K.R., Osinnikov I.V. (2022). Study of the Structure and Mechanical Properties after Electrical Discharge Machining with Composite Electrode Tools. Materials.

[31] Konieczny M., Szwed B., Mola R. Diffusion bonding and transient liquid phase joining of titanium to AlSi 304 stainless steel with analuminum interlayer. Proceedings of the Metal 2015: 24th International Conference on Metallurgy and Materials.

[32] Masuzawa T., Yamaguchi M., Fujino M. (2005). Surface Finishing of Micro pins Produced by WEDG. CIRP Ann.-Manuf. Technol..

[33] Guu Y.H. (2005). AFM surface imaging of AISI D2 tool steel machined by the EDM process. Appl. Surf. Sci..

[34] Han F., Jiang J., Yu D. (2007). Influence of discharge current on machined surfaces by thermoanalysis in finish cut of WEDM. Int. J. Mach. Tools Manuf..

[35] Mohanty C.P., Mahapatra S.S., Singh M.R. (2017). An intelligent approach to optimize the EDM process parameters using utility concept and QPSO algorithm. Eng. Sci. Technol. Int. J..

[36] Świercz R. (2019). Modeling and Optimization of Electrical Discharge Machining of Difficult-to-Machine Materials.

[37] Mohapatra J., Xing M., Elkins J., Liu P.J. (2020). Hard and semi-hard magnetic materials based on cobalt and cobalt alloys. J. Alloys Compd..

[38] Przybyłowicz K. (2007). Metalozanawstwo.

[39] Dabade U.A., Karidkar S.S. (2016). Analysis of Response Variables in WEDM of Inconel 718 Using Taguchi Technique. Procedia CIRP.

[40] Adamczak S., Zmarzły P. (2019). Research of the influence of the 2D and 3D surface roughness parameters of bearing race ways on the vibration level. J. Phys. Conf. Ser..

[41] Adamczak S., Miko E., Cus F. (2009). A model of surface roughness constitution in the metal cutting process applying tools with defined stereometry. Stroj. J. Mech. Eng..

[42] (2010). Geometrical Product Specifications (GPS)—Surface Texture: Areal—Part 6: Classification of Methods for Measuring Surface Texture.

